# Position statement on macroprolactinemia from the Department of Neuroendocrinology of the Brazilian Society of Endocrinology and Metabolism (SBEM) and the Brazilian Society of Clinical Pathology/Laboratory Medicine (SBPC/ML)

**DOI:** 10.20945/2359-4292-2025-0152

**Published:** 2025-10-28

**Authors:** Andrea Glezer, Paula Condé Lamparelli Elias, Vania dos Santos Nunes Nogueira, Heraldo Mendes Garmes, Leandro Kasuki, Guilherme Alcides Flôres Soares Rollin, Manoel Ricardo Alves Martins, Adriana Caschera Leme, Pedro Saddi Rosa, Luciana Ansaneli Naves, Marcelo Cidade Batista

**Affiliations:** 1 Unidade de Neuroendocrinologia, Hospital das Clínicas, Universidade de São Paulo, São Paulo, SP, Brasil; 2 Departamento de Clínica Médica, Faculdade de Medicina de Ribeirão Preto, Universidade de São Paulo, Ribeirão Preto, SP, Brasil; 3 Universidade Estadual Paulista, Faculdade de Medicina, São Paulo, SP, Brasil; 4 Disciplina de Endocrinologia e Metabologia, Departamento de Clínica Médica, Faculdade de Ciências Médicas, Universidade Estadual de Campinas, Campinas, SP, Brasil; 5 Centro de Pesquisa em Neuroendocrinologia, Hospital Universitário Clementino Fraga Filho/Universidade Federal do Rio de Janeiro e Unidade de Neuroendócrina do Instituto Estadual do Cérebro Paulo Niemeyer, Rio de Janeiro, RJ, Brasil; 6 Serviço de Endocrinologia e Nutrologia, Hospital Moinhos de Vento, Porto Alegre, RS, Brasil; 7 Departamento de Medicina Clínica e Núcleo de Pesquisa e Desenvolvimento de Medicamentos, Universidade Federal do Ceará, Fortaleza, CE, Brasil; 8 Laboratório Clínico, Hospital Israelita Albert Einstein, São Paulo, SP, Brasil; 9 Fleury Medicina e Saúde, São Paulo, SP, Brasil; 10 Serviço de Endocrinologia, Hospital Universitário, Universidade de Brasília, DF, Brasil Grupo Sabin, Brasília, DF, Brasil

## Abstract

Measurement of serum prolactin levels is a common practice in clinical settings, particularly among women of reproductive age. In cases of hyperprolactinemia, identifying macroprolactinemia can help prevent unnecessary investigation and inappropriate treatments. This Position Statement, jointly prepared by the Brazilian Society of Endocrinology and Metabolism (SBEM) and the Brazilian Society of Clinical Pathology/Laboratory Medicine (SBPC/ML), addresses several aspects of macroprolactinemia relevant to clinical practice – including concepts, definitions, epidemiological aspects, measurement techniques, and the role of screening – and discusses some clinical dilemmas.

## TARGET AUDIENCE

Measurement of serum prolactin levels is a common practice in clinical settings, particularly among women of reproductive age. Hyperprolactinemia, characterized by elevated serum prolactin levels outside pregnancy and the postpartum period, is a prevalent endocrine disorder that requires accurate diagnosis and treatment (^1,2^). In this context, it is crucial to identify cases of increased levels of macroprolactin, as its undetected presence can result in misdiagnosis and inappropriate management of hyperprolactinemia (^3,4^). 

This Position Statement, developed collaboratively by the Brazilian Society of Endocrinology and Metabolism (SBEM) and the Brazilian Society of Clinical Pathology/Laboratory Medicine (SBPC/ML), examines several aspects of macroprolactinemia that are crucial to clinical practice. It covers essential concepts and definitions, epidemiological insights, measurement techniques, and the significance of screening, as well as various clinical dilemmas. This is an exceptionally pertinent topic for endocrinologists, general practitioners, gynecologists, urologists, and professionals deeply involved in clinical pathology and laboratory medicine.

## OBJECTIVES

Macroprolactin may cross-react with prolactin to varying degrees in most immunoassays (^5^). Because it has little biological activity and no clinical impact, its detection can help avoid additional investigations or treatments, which are only recommended for patients with hyperprolactinemia caused by increased levels of monomeric (bioactive) prolactin (^6^). The lack of adequate knowledge about macroprolactin in the context of hyperprolactinemia results in unnecessary investigations and treatments, burdening the healthcare system and posing risks to patients. Thus, this Position Statement aims to disseminate knowledge about macroprolactin, with guidelines and recommendations on when to request, interpret, and monitor patients with macroprolactinemia.

## DEFINITION OF MACROPROLACTIN AND MACROPROLACTINEMIA

The primary role of prolactin is to promote mammary gland development and lactation in humans and animal models (^7^). During late pregnancy, estrogen-induced lactotroph hyperplasia increases prolactin levels, which in turn lead to mammary epithelial proliferation, differentiation, and milk production postpartum. In animal models, prolactin exerts a complex control over the reproductive axis by modulating hypothalamic dopamine and kisspeptin neuron activity, thereby influencing gonadotropin-releasing hormone (GnRH) and downstream luteinizing hormone (LH) and follicle-stimulating hormone (FSH) secretion (^8^). Increased prolactin levels disrupt reproductive homeostasis, causing hypogonadotropic hypogonadism, menstrual irregularities, galactorrhea, and infertility – symptoms classically associated with hyperprolactinemia (^9^). 

Prolactin is encoded by a single 10-kb gene located on chromosome 6, composed of five coding exons, one non-coding exon, and four introns (^6^). This hormone derives from a 227-amino acid prohormone that is cleaved at the 28-amino acid signal peptide, resulting in a final compound of 23 kDa with 199 amino acids, organized in a single chain with three disulfide bridges between six cysteine residues (^10^). The monomeric prolactin has a molecular weight of 23 kDa and corresponds to 85%-95% of the synthesized prolactin. Biochemical phenomena may produce other forms, resulting in variants with higher or lower molecular weight (**Table 1**). Dimerization, polymerization, or aggregation of prolactin with binding proteins and immunoglobulins results in molecules of higher molecular weight and low biological activity. Most immunoassays used to measure prolactin cross-react with these molecules, posing a major dilemma in therapeutic decision-making. The most prevalent forms are dimeric prolactin (“big prolactin”) and macroprolactin (“big-big prolactin”).

**Table 1:** Aggregates and isoforms of monomeric prolactin, which generate compounds with analytical immunoreactivity and lower bioactivity

Nomenclature		Molecular weight	Biological activity
Monomeric	Bioactive prolactin	23 kDa	Present
Aggregation of immunoglobulins or binding proteins			
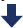			
High molecular weight proteins			
**Nomenclature**		Molecular weight	Biological activity
Dimeric	“Big prolactin”	40-60 kDa	Low/absent
Polymeric	“Big-big prolactin”	>100-150 kDa	Low/absent
	IgG 0%-8%		
	IgA 0%-2%		
	Others 0%-3%		

Adapted from 6,17,18.

Macroprolactin has a molecular weight exceeding 150 kDa and minimal biological activity. It consists of monomeric prolactin bound primarily to immunoglobulin G (IgG), accounting for 87% of the complex, and to a lesser extent to immunoglobulin A (IgA). Most of the bound IgG (about 67%) consists of anti-prolactin autoantibodies, with the remainder composed of nonspecific heterophilic antibodies (^11^). Although the pathogenesis of macroprolactinemia remains unclear, some authors suggest that genetic predisposition to post-translational modifications of monomeric prolactin, such as glycosylation, phosphorylation, or deamidation, may expose new epitopes that are not recognized as self-antigens and trigger the production of autoantibodies (^3,12,13^). A minor fraction of macroprolactin is formed by glycosylation, aggregation, and covalent or non-covalent binding of prolactin molecules (^14^). 

The diagnosis of macroprolactinemia is established when more than 60% of circulating prolactin is composed of macroprolactin (^4^). The gold-standard method for measuring macroprolactin is gel filtration chromatography (GFC). However, this method is cumbersome, time-consuming, and expensive for use by clinical laboratories. A simpler and more practical technique is to precipitate the sample with polyethylene glycol (PEG), centrifuge it, and remeasure prolactin in the supernatant. Since PEG precipitates molecules or aggregates with high molecular weight, the fraction remaining in the supernatant (percentage [%] recovery) corresponds to the monomeric, bioactive 23 kDa prolactin. When this fraction is below 40% of the initial prolactin level, it strongly indicates the predominance of macroprolactin in the sample (^15,16^).

Macroprolactin may be solely responsible for hyperprolactinemia (*e.g.*, isolated macroprolactinemia), in which case monomeric prolactin levels are normal. Patients with isolated macroprolactinemia do not require further diagnostic investigation or treatment. In contrast, several patients only have increased concentrations of monomeric prolactin (monomeric or true hyperprolactinemia), while others present with elevated concentrations of both macroprolactin and monomeric prolactin. These two conditions require a more thorough diagnostic evaluation to identify the real cause of the monomeric hyperprolactinemia before choosing the best therapeutic approach (^15,16^). 

## PREVALENCE AND POPULATION STUDIES

The prevalence of macroprolactinemia varies depending on the immunoassay used to measure prolactin, the different reference intervals for diagnosing hyperprolactinemia, and the cutoff values for post-PEG % recovery used to define macroprolactinemia. A recent meta-analysis involving 16,951 patients from 27 countries found great heterogeneity among the analyzed studies. Most studies used an immunometric chemiluminescent assay (ICMA) to measure prolactin. The mean prevalence of macroprolactinemia among patients with hyperprolactinemia was 18.9% (95% confidence interval 15.8%-22.1%), ranging from 0% to 55.6% across studies. It was not possible to compare the differences in prevalence between the sexes (^2^).

In a recent Brazilian study involving 770 patients diagnosed with hyperprolactinemia, macroprolactin testing using PEG precipitation was performed in idiopathic cases and was positive in 28.3%. Although most patients in the study had serum prolactin levels below 100 ng/mL (mean 88.70 ng/mL), the values ranged from 40 ng/mL to 490 ng/mL (^19^). In a previous multicenter Brazilian study including 1,234 patients with hyperprolactinemia, macroprolactinemia was diagnosed in 9.3%, and serum prolactin levels were 119.5±112.9 ng/mL, ranging from 32.5 ng/mL to 404 ng/mL (^20^). In case series of patients with macroprolactinemia, the median prolactin levels ranged between 46.2 ng/mL (^21^) and 129.12 ng/mL (^22^). Most cases of macroprolactinemia have prolactin levels below 200 ng/mL (^23^). 

## *IN VITRO* AND *IN VIVO* BIOLOGICAL ACTIVITY: CLINICAL IMPLICATIONS

### *In vitro* biological activity

The biological activity of macroprolactin has been previously studied \*in vitro* (^24^) with conflicting results. One reason for the discrepancy is that monomeric prolactin may dissociate from the IgG molecule in the sample, resulting in some biological activity (^25^). However, in two studies with cell cultures expressing the human prolactin receptor, macroprolactin did not show biological activity, while monomeric prolactin was bioactive (^26,27^). It is worth noting that anti-prolactin autoantibodies and prolactin receptors bind to similar regions of the prolactin molecule; therefore, autoantibodies may compete with the binding of the prolactin molecule to its receptors, resulting in low activity (^11^). Thus, several data point to the low bioavailability and reduced bioactivity of macroprolactin (^12^).

### *In vivo* biological activity

It is generally accepted that macroprolactin has a reduced biological activity *in vivo*. Due to its high molecular weight, macroprolactin does not cross the blood capillary barrier, limiting its activity *in vivo* (^13^). The first cases of macroprolactinemia reported in the literature were identified in asymptomatic individuals (^28^). Several subsequent case series have reported a lower overall prevalence of signs and symptoms of prolactin excess in patients with macroprolactinemia than in those with increased levels of monomeric prolactin. About 60% of individuals with macroprolactinemia have no signs or symptoms of prolactin excess [which frequently include galactorrhea, menstrual irregularity, and amenorrhea (^28^)], although the clinical presentation can overlap between patients with increased macroprolactin and monomeric prolactin levels, particularly in terms of infertility (^13^).

Several factors may contribute to the occurrence of symptoms in patients with macroprolactinemia. First, symptoms commonly associated with hyperprolactinemia can trigger laboratory investigation, but may be only a coincidental finding in patients with macroprolactinemia (^13^). Second, conditions such as polycystic ovary syndrome (^29,30^), idiopathic galactorrhea, and psychogenic erectile dysfunction (^31^) can be found in individuals with macroprolactinemia (^29,30^). Third, some patients may have macroprolactinemia associated with increased levels of monomeric prolactin, which is the cause of the clinical manifestations (^32^). 

A compilation of 11 case series (^5,21,22,32-39^) involving over 5,000 patients with hyperprolactinemia and more than 1,000 with macroprolactinemia showed ranges in the frequency of menstrual irregularity, galactorrhea, and infertility at 14%-59%, 2%-46%, and 5%-68%, respectively. These clinical findings were more common among patients with hyperprolactinemia and no macroprolactinemia. Magnetic resonance imaging (MRI) was performed in 10 case series, and normal results were much more common in the macroprolactinemia group (^5,22,33-41^). **Table 2** summarizes the studies cited and is found in the **Supplementary Material**. Overall, these studies showed that symptoms related to hyperprolactinemia are substantially less common in individuals with macroprolactinemia, while normal sellar imaging is much more frequent in this group, reinforcing the benign nature of the condition.

In summary, studies show that macroprolactin exhibits low biological activity both *in vitro* and \*in vivo*. In individuals with isolated macroprolactinemia, sellar imaging is not recommended, and, if associated symptoms are present, other causes should be considered – especially polycystic ovary syndrome in women of childbearing age. In contrast, in patients with macroprolactinemia associated with monomeric hyperprolactinemia, it is important to identify the underlying cause. 

## METHODS, SCREENING, AND THE GOLD-STANDARD TECHNIQUE FOR DETECTING MACROPROLACTIN

Initial reports published in the 1980s documented elevated levels of a prolactin variant with high molecular weight in women with sustained hyperprolactinemia and preserved fertility; measurement of prolactin in these studies was performed using GFC, which is currently considered the gold-standard method for measuring macroprolactin (^42,43^). In 1985, Jackson and cols. introduced the term macroprolactinemia to describe this prolactin variant and characterized the molecule as having no biological activity (^44^). 

Despite its low biological activity, macroprolactin is detectable in automated immunometric assays commonly used for measuring prolactin, although reactivity may vary from high to low depending on the assay used (^42,43^). Since GFC is a laborious, expensive, and time-consuming method in clinical practice, the PEG precipitation technique has become widely used as a simple and convenient alternative for screening for macroprolactin (^16,45,46^). Acting as an inert sponge, PEG reduces the solvent’s availability. This leads to an increase in protein concentration until its solubility is exceeded and precipitation occurs. When applied to serum, PEG mainly precipitates immunoglobulins and immunoglobulin complexes.

It is important to note that with PEG, precipitation of IgA occurs only partially, and in the rare cases of macroprolactinemia due to IgA binding, macroprolactin may be undetected (^45^). In contrast, PEG also precipitates the rare polymers of prolactin that do not contain immunoglobulin, as well as the big prolactin component of total serum immunoreactive prolactin (^47^). Additionally, PEG can also precipitate part of the monomeric prolactin in the sample, which is partly determined by serum gamma globulin concentrations. Elevated serum globulin levels may increase the amount of monomeric prolactin precipitated by PEG, resulting in a false estimate of monomeric prolactin and the erroneous impression that macroprolactin is present. Therefore, PEG precipitation test results should be interpreted with caution in patients with elevated serum globulin concentrations (^47^).

**Figure 1:**
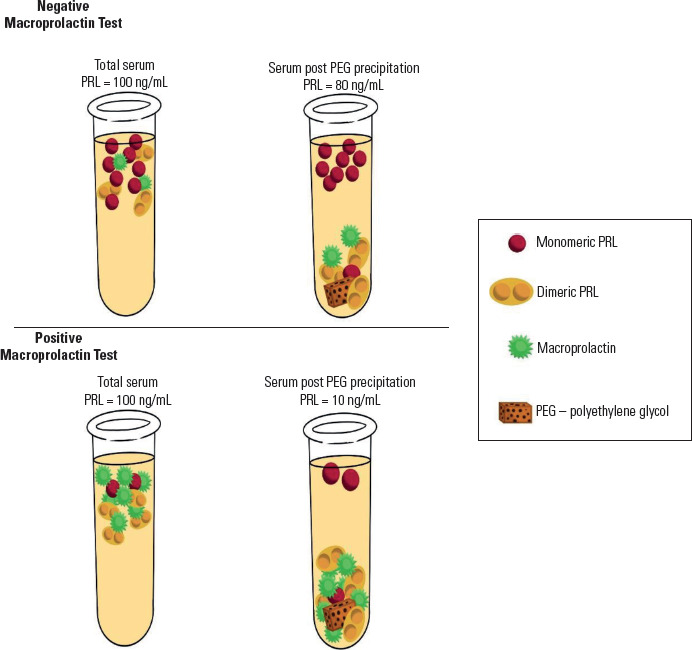
Schematic representation of macroprolactin testing using polyethylene glycol (PEG) in samples with and without macroprolactinemia.

Once added to the hyperprolactinemic sample, PEG promotes the precipitation of macroprolactin after centrifugation, leaving behind residual monomeric prolactin in the supernatant, which can then be remeasured (**Figure 1**). The ratio of prolactin concentration measured in the sample before and after PEG precipitation estimates the fraction of monomeric and biologically active prolactin (% recovery). A suggested protocol for the PEG precipitation test is provided in the **Supplementary Material**.

Monomeric prolactin recovered after PEG treatment is usually expressed as a percentage of the total baseline prolactin (% recovery). When macroprolactin is the predominant form, the % recovery usually falls below 40%. Intermediate values between 40% and 60% are often classified as indeterminate, with GFC analysis recommended for a definitive diagnosis (^45^). Values greater than 60% suggest the absence of macroprolactinemia.

Importantly, the % recovery can only detect whether excess macroprolactin is present, but does not provide information on the concomitant presence of increased monomeric prolactin levels. In many patients, increased macroprolactin and monomeric prolactin levels may occur concurrently, requiring investigation into the potential causes of the latter. Therefore, from a clinical standpoint, the macroprolactin report should include not only the % recovery but also the basal and post-PEG prolactin values, along with the respective reference intervals specific to the method used by the laboratory. A suggested macroprolactin test report is provided in **Box 1**. 

It is noteworthy that reference intervals for post-PEG prolactin are generally lower than those of basal prolactin. This occurs because a small fraction (about 20%-25%) of monomeric prolactin is precipitated by PEG, along with the higher molecular weight forms (^48-50^). Two published studies provide reference intervals for basal and post-PEG prolactin for the main immunoassays used by clinical laboratories (^48,49^).

Box 1 Suggested macroprolactin test report

- Baseline prolactin: XXX.X ng/mL (laboratory-specific reference interval).
- Post-PEG precipitation prolactin: YYY.Y ng/mL (laboratory-specific reference interval).
- Post-PEG precipitation % recovery: XX% (<40%: macroprolactinemia present; >60%: macroprolactinemia absent; 40%-60%: indeterminate; gel filtration chromatography analysis suggested).
- Interpretation:
  ■ % recovery < 40% and normal post-PEG prolactin: isolated macroprolactinemia.
  ■ % recovery > 60% and elevated post-PEG prolactin: isolated monomeric hyperprolactinemia.
  ■ % recovery < 40% and elevated post-PEG prolactin: macroprolactinemia associated with monomeric hyperprolactinemia.

## WHEN TO SCREEN FOR MACROPROLACTIN

The two following strategies are proposed for screening macroprolactin (^51^).

### Strategy #1 – Physician-directed macroprolactin screening (**Figure 2**)

**Figure 2:**
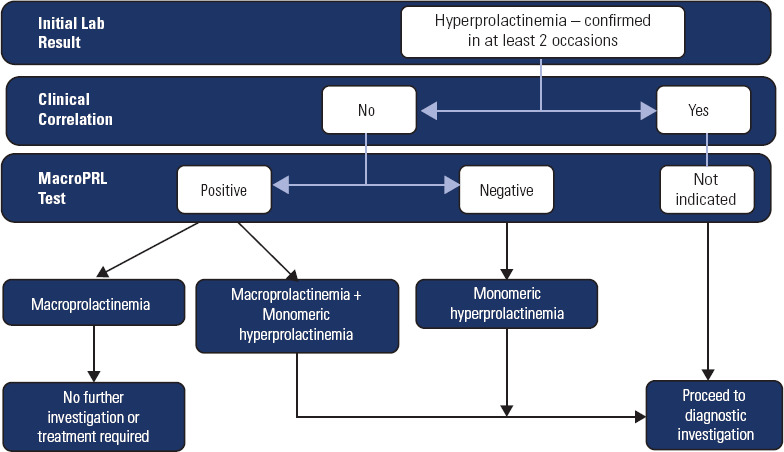
Flowchart for the investigation of macroprolactinemia according to physician-directed macroprolactin screening strategy.

In an ideal setting, the best approach to investigating hyperprolactinemia and macroprolactinemia is: (i) the physician requests an initial prolactin measurement, particularly in patients with clinical symptoms of hyperprolactinemia and/or infertility; (ii) if elevated, the laboratory provides a report with the prolactin level and a note suggesting confirmation of the result with another sample and macroprolactin testing. The second sample should preferably be collected in the morning (within 2-3 hours after waking), after fasting for at least 8 hours, and avoiding vigorous physical exercise 30 minutes before (^52^); (iii) once hyperprolactinemia is confirmed, the laboratory performs macroprolactin testing on the repeat sample, if requested by the physician. This is probably the best strategy for screening for macroprolactin, because it relies on the patient’s clinical presentation, which is usually only available to the patient’s doctor. It also allows the laboratory to bill for the testing more easily, since it will be specified in the doctor’s order. However, many physicians may not be fully aware of macroprolactin or may overlook notes in lab reports, so they might not request macroprolactin testing (^51^). As a result, they may interpret all cases of hyperprolactinemia as being due to pituitary dysfunction and order expensive tests such as sellar MRI and/or recommend drug or surgical treatments – all of which are unnecessary in cases of macroprolactinemia.

### Strategy #2 – Laboratory-directed macroprolactin screening

In this strategy, the laboratory directs all samples with elevated prolactin levels to macroprolactin testing as a reflex test (^14^). In general, samples from pregnant or lactating women, patients with confirmed prolactinoma, and/or those taking medications that may increase prolactin levels (data often unavailable to the laboratory) are excluded from macroprolactin testing. The main advantage of this strategy is that it detects all cases of macroprolactinemia. Its main disadvantages include the fact that (i) macroprolactin testing will often be performed unnecessarily, since it will not be based on the patient’s clinical presentation, and (ii) some laboratories may find it difficult to bill for the test, as it is not included in the medical order. However, considering that PEG testing is a relatively simple and inexpensive procedure compared to sellar MRI, long-term medical treatment, or pituitary surgery, it may well be worth the extra cost of testing to avoid undetected cases of macroprolactin, particularly in real-world settings where physicians may not be aware of macroprolactinemia. The cutoff level that triggers automatic macroprolactin testing should be the upper limit of the reference interval for the assay used by the laboratory (^50^). Using empirical values based on older methods (such as 30.0 ng/mL, 32.9 ng/mL, or 47.0 ng/mL) should be avoided, as applying these cutoff values can result in missing many macroprolactin cases. Recent studies have shown that macroprolactinemia is more common in women with mildly elevated prolactin levels (*e.g.*, between 23.6 ng/mL and 33.1 ng/mL) when measured using the Elecsys Roche assay (^53^).

In summary, laboratories may choose either of these two strategies, taking into account local technical and financial aspects. In the United States, the first strategy is the most widely used (^53^), while in Europe – especially in the United Kingdom and Spain – the second strategy is preferred (^14^). 

## WHEN AND HOW TO FOLLOW UP PATIENTS WITH MACROPROLACTINEMIA

Several studies published over the past 25 years have followed up patients with macroprolactinemia to determine whether this condition is benign and remains stable over time, thus not requiring monitoring. The first study, including 106 patients followed for 2-7 years, found that, on average, prolactin levels remained stable. There was no consistent change over time, although sporadic fluctuations were observed in several patients, sometimes reaching amplitudes greater than five times (^36^). Other studies, including up to 50 subjects with macroprolactinemia followed for more than 10 years, also reported no progression or manifestation of symptoms related to autoimmunity or hyperprolactinemia (headache, visual changes, menstrual irregularity, galactorrhea, and infertility), nor any changes in imaging exams. Of note, spontaneous pregnancies occurred during follow-up (^36,54,55^). Recent data from a series of 790 patients followed for 4 years also demonstrated 96% agreement between positive and negative results of serial macroprolactin tests. The authors recommended repeating macroprolactin testing only when prolactin levels increase significantly over time (^56^). These data were confirmed in another study in which 465 patients underwent 1,437 macroprolactin tests (^57^). In summary, these findings support the recommendation against repeating prolactin measurement or macroprolactin screening in the absence of changes in clinical manifestations. 

## COSTS OF MACROPROLACTIN MEASUREMENT VERSUS UNNECESSARY TESTS AND TREATMENTS

Patients with macroprolactinemia often do not have increased levels of monomeric (bioactive) prolactin; thus, this condition primarily represents a laboratory pitfall. Therefore, macroprolactin screening is necessary to ensure that these patients do not undergo additional and extensive diagnostic evaluation or receive unnecessary treatment with dopamine agonists or neurosurgery. This not only exposes them to potential adverse events but also incurs unnecessary costs. On a large scale, this practice can overburden both public and private healthcare systems.

Considering the prevalence of hyperprolactinemia treated with dopamine agonists as 30 cases per 100,000 individuals (^19,58,59^), the frequency of asymptomatic macroprolactinemia at 18% (^19,58,59^), and data obtained from the Brazilian IBGE demographic census, the additional cost for the Brazilian Unified Health System (SUS) due to this unnecessary treatment was estimated at approximately BRL 5,000,000 (about USD 900,000). For this cost analysis, data were sourced from the Management System for the Table of Procedures, Medications, and Orthoses, Prostheses and Special Materials (SIGTAP) of the SUS. The price of cabergoline was obtained from the Health Price Database, with calculation based on a dosage of 1 mg per week over a 2-year period (**Table 3, Supplementary Material**).

Our final recommendations are summarized in **Box 2**.

Box 2 Final recommendations

■ The laboratory may only proceed with macroprolactin testing when requested by the clinician or perform it on all samples with prolactin levels above the specific reference interval for the immunoassay used.
■ Although GFC is the gold-standard method for macroprolactin testing, it is not highly feasible in clinical practice. Macroprolactin screening with PEG precipitation is a simpler and more practical alternative, and is the most commonly used method in clinical laboratories for this purpose.
■ In patients with isolated macroprolactinemia and no change in clinical presentation over time, repeating prolactin measurements or macroprolactin testing is not necessary.
■ Investigation and treatment are unnecessary for asymptomatic individuals with isolated macroprolactinemia and may expose them to adverse effects and burden healthcare systems.

In conclusion, prolactin can undergo various post-translational processes, including dimerization, which produces dimeric prolactin, and polymerization or aggregation with binding proteins and/or immunoglobulins, resulting in macroprolactin. These forms are characterized by high molecular weight and low biological activity. The prevalence of macroprolactinemia, defined as the presence of the main circulating prolactin variant as macroprolactin, is approximately 20% among patients with hyperprolactinemia. Macroprolactin has low biological activity, and macroprolactinemia is a benign condition. 

Evaluation of prolactin should be triggered by clinical findings (hypogonadism, galactorrhea, infertility, and gynecomastia) and/or imaging findings (pituitary incidentaloma). In the presence of hyperprolactinemia and absence of clinical manifestations, macroprolactinemia should be ruled out. In contrast, in the presence of signs and symptoms possibly related to hyperprolactinemia in a patient with macroprolactinemia and normal levels of monomeric prolactin, other causes for the clinical findings should be evaluated. 

Clinicians and laboratories should be aware of macroprolactinemia and establish standardized protocols to screen for macroprolactin, ensuring that results are easily interpretable and improving the clinical-laboratory interface for the patient’s benefit.

## Data Availability

datasets related to this article will be available upon request to the corresponding author.

## Supplementary MATERIALS

**Supplementary Table 1:** Suggested protocol for the polyethylene glycol (PEG) precipitation test

**1. Preparation of the PEG solution** Dissolve 25 g of PEG 6000 in approximately 60 mL of distilled or deionized water at 18-25 °C, using a magnetic stirrer for 15 minutes. Subsequently, complete the volume to 100 mL with the same type of water.
**2. Sample treatment** Homogenize the sample (minimum 180 µL) with the PEG solution in a 1:1 ratio for approximately 1 minute using a vortex mixer. Within 1-30 minutes, centrifuge the mixture at 1,500-3,000 g for 5 minutes.
**3. Analysis of the supernatant** After centrifugation, re-assay the prolactin in the supernatant. This fraction represents the monomeric prolactin (biologically active), distinguishing it from the macroprolactin.
**4. Calculation of percentage (%) recovery** Use the following formula to calculate the % recovery, using the multiplication factor (^2^) to account for the 1:1 dilution of the initial sample (valid only for this dilution): **(Prolactin measured in the supernatant × 2) ÷ total baseline prolactin)**

**Supplementary Table 2:** Case series of macroprolactinemic patients, describing their clinical and imaging findings, compared patients with hyperprolactinemia

Author, year	Hyperprolactinemia (n)	Monomeric prolactin or Macroprolactin n (%)	Clinical and imaging findings % (n when available)				
			Erectile dysfunction	Menstrual irregularity	Infertility	Galactorrhea	Sellar imaging
Leslie and cols., 2001 (^33^)	1,225	Monoprolactin 903 (74%)	N/A	N/A	N/A	N/A	N/A
		Macroprolactin 322 (26%)	N/A	44% (24/55)	24% (13/55)	4% (2/55)	7.2% (MIC)
Hauache and cols., 2002 (^34^)	113	Monoprolactin 61 (54%)	N/A	N/A	N/A	N/A	25% (Normal)
		Macroprolactin 52 (46%)	N/A	36% (10/28)	7% (2/28)	25 (7/28)	79% (Normal)
Strachan and cols., 2003 (^35^)	273	Monoprolactin 215 (79%)	N/A	N/A	N/A	N/A	N/A
		Macroprolactin 58 (21%)	71%	20%	11%	14%	14% (MIC) 3% (MAC)
Vallette-Kasic and cols., 2002 (^36^)	1,106	Monoprolactin 1,000 (90%)	N/A	49%	70%	66%	78.6% (Abnormal)
		Macroprolactin 106 (10%)		39%^a^	68%	46%^a^	17% (Abnormal)
Suliman and cols., 2003 (^5^)*	63	Monoprolactin 42 (67%)	N/A	84%	29%	63%	34% (Abnormal)
		Macroprolactin 21 (33%)	N/A	57%^a^	8%	29%^a^	15% (Abnormal)
Gibney and cols., 2005 (^32^)*	2,089	Monoprolactin 1636 (78%) (100 evaluated)	N/A	73%	7%	54%	15% (Abnormal)
		Macroprolactin 453 (22%) (32 evaluated)	N/A	59%^a^	22%	22%^a^	2% (Abnormal)
Alfonso and cols., 2006 (^37^)*	40	Monoprolactin 22 (55%)	60%	47%	24%	41%	57% (MIC)
		Macroprolactin (45%)	78%	56%	11%	11%	56% (Normal)
Donadio and cols., 2007 (^38^)	135	Monoprolactin 78 (58%)	54%	71%	N/A	40%	19% (Normal)
		Macroprolactin 57 (42%)	27%	45%^a^	N/A	20%^a^	46% (Normal)^a^
Isik and cols., 2012 (^39^)*	337	Monoprolactin 249 (74%)	72%	54%	11%	57%	81% (Abnormal)
		Macroprolactin 88 (26%)	50%	35%	5%	39%^a^	66% (Abnormal)^a^
Tamer and cols., 2012 (^22^)*	161	Monoprolactin 101 (63%)	N/A	31%	21%	30%	55% (Abnormal)
		Macroprolactin 60 (37%)	N/A	18%	N/A	15%^a^	27% (Abnormal)^a^
Thirunavakkarasu and cols., 2013 (^21^)	183	Monoprolactin 162 (89%)	N/A	46%	N/A	30%	N/A
		Macroprolactin 21 (11%)	N/A	14%^a^	N/A	5%a	N/A

* Cases of macroprolactinemia associated with monomeric hyperprolactinemia were excluded.

a Significant difference between macroprolactinemia and monomeric hyperprolactinemia. Abbreviations: hyperprolactin, total hyperprolactinemia; monoprolactin, monomeric hyperprolactinemia; macroprolactin, macroprolactinemia; MIC: microadenoma; MAC: macroadenoma; N/A: not available.

**Supplementary Table 3:** Estimation of the direct medical cost for managing hyperprolactinemia, including the measurement of macroprolactinemia, in women with asymptomatic hyperprolactinemia from the perspective of the Brazilian Unified Health System (SUS)

**IBGE Census in 2022: Brazilian population between the ages of 20 and 64.9 years**		
Women	65,277,172	IBGE*
Men	61,129,280	IBGE*
**Prevalence of hyperprolactinemia treated with dopamine agonists (^1^)**		Kars M et al.
Women	0.000295	
**Prevalence of Macroprolactinemia (^2^)**	0.28	Vilar L et al.
Total number of women with hyperprolactinemia	19,256.77	
**Prevalence of Asymptomatic Macroprolactinemia (^3^)**	0.63	Vilar L et al.
Total number of women with asymptomatic macroprolactinemia	3,396.89	
**Cost of treatment of idiopathic hyperprolactinemia and/or microadenoma in the SUS**		
02.07.01.007-2 - Magnetic resonance imaging of the sella turcica	BRL 268.75	SIGTAP**
03.01.01.007-2 - Medical consultation in specialized care	BRL 10.00	SIGTAP**
02.02.01.064-3 - Aspartate aminotransferase (AST) measurement	BRL 2.01	SIGTAP**
02.02.01.065-1 - Alanine aminotransferase (ALT) measurement	BRL 2.01	SIGTAP**
02.02.01.031-7 - Creatinine measurement	BRL 1.85	SIGTAP**
02.02.06.030-6 - Prolactin measurement	BRL 10.15	SIGTAP**
Total in exams over 2 years	BRL 104.08	
**02.02.06.047-0 - Macroprolactin testing**	BRL 12.15	SIGTAP**
Cost of cabergoline 0.5 mg tablet	BRL 5.30	BPS***
Number of cabergoline pills over 2 years (two pills per week)	208	
Total cost per patient of cabergoline over 2 years	BRL 1,102.40	
Cost per patient of idiopathic hyperprolactinemia/microadenoma treatment with cabergoline 1 mg/week	BRL 1,475.23	
**Additional SUS billing for the treatment of asymptomatic macroprolactinemia**	BRL 4,969,926.91	

* https://www.ibge.gov.br/estatisticas/sociais/populacao/22827-censo-demografico-2022.html?edicao=38166&t=resultados

** http://sigtap.datasus.gov.br/tabela-unificada/app/sec/inicio.jsp

*** https://infoms.saude.gov.br/extensions/SEIDIGI_DEMAS_BPS/SEIDIGI_DEMAS_BPS.html
